# Immune response to recombinant *Burkholderia pseudomallei* FliC

**DOI:** 10.1371/journal.pone.0198906

**Published:** 2018-06-14

**Authors:** Sirikamon Koosakulnirand, Phornpun Phokrai, Kemajittra Jenjaroen, Rosemary A. Roberts, Pongsak Utaisincharoen, Susanna J. Dunachie, Paul J. Brett, Mary N. Burtnick, Narisara Chantratita

**Affiliations:** 1 Department of Microbiology, Faculty of Science, Mahidol University, Bangkok, Thailand; 2 Department of Microbiology and Immunology, Faculty of Tropical Medicine, Mahidol University, Bangkok, Thailand; 3 Mahidol-Oxford Tropical Medicine Research Unit, Mahidol University, Bangkok, Thailand; 4 Department of Microbiology and Immunology, University of South Alabama, Mobile, AL, United States of America; 5 Center for Tropical Medicine and Global Health, University of Oxford, Oxford, United Kingdom; University of Toledo College of Medicine and Life Sciences, UNITED STATES

## Abstract

*Burkholderia pseudomallei* is a flagellated Gram-negative bacterium which is the causative agent of melioidosis. The disease poses a major public health problem in tropical regions and diabetes is a major risk factor. The high mortality rate of melioidosis is associated with severe sepsis which involves the overwhelming production of pro-inflammatory cytokines. Bacterial flagellar protein (flagellin) activates Toll-like receptor 5 (TLR5)-mediated innate immune signaling pathways and induces adaptive immune response. However, previous studies of TLR5 signaling in melioidosis have been performed using recombinant flagellin from *Salmonella* Typhimurium instead of *B*. *pseudomallei*. This study aimed to investigate human innate immune response and antibody response against a recombinant *B*. *pseudomallei* flagellin (rFliC). We prepared *B*. *pseudomallei* rFliC and used it to stimulate HEK-Blue^TM^-hTLR5 and THP1-Dual^TM^ cells to assess TLR5 activation. Subsequently, whole blood stimulation assays with rFliC were performed *ex vivo*. TLR5-flagellin interactions trigger activation of transcription factor NF-κB in HEK-Blue^TM^-hTLR5 cells. Pro-inflammatory cytokine (IL-1β, IL-6, and TNF-α) productions from whole blood in response to rFliC differed between fourteen healthy individuals. The levels of these cytokines changed in a dose and time-dependent manner. ELISA was used to determine rFliC-specific antibodies in serum samples from different groups of melioidosis patients and healthy subjects. IgG antibody to rFliC in melioidosis patients with diabetes were higher compared with non-diabetic patients. Our results show that *B*. *pseudomallei* flagellin is a potent immune stimulator and that the immune responses to rFliC are different among individuals. This may provide valuable insights toward the potential use of rFliC in vaccine development.

## Introduction

*Burkholderia pseudomallei* is a flagellated, environmental, Gram-negative bacterium which is the causative agent of melioidosis, a community-acquired infection that is endemic in Southeast Asia and northern Australia. The mortality rates of melioidosis vary between countries ranging from 14% in Australia to 40% in northeast Thailand [1–3]. Melioidosis is commonly associated with host risk factors, the majority of which is diabetes presenting in 23% to 60% of patients and type II diabetes is common. Clinical symptoms in melioidosis patients are varied, but often present as pneumonia, bacteremia, genitourinary infections, skin infections and abscesses in internal organs. In many cases, melioidosis can present as chronic and persistent infections [1, 3]. The high mortality rate of melioidosis is attributed to bacteremia and severe sepsis, which involves the overwhelming production of pro-inflammatory cytokines.

Understanding the host immune response to *B*. *pseudomallei* infection is critical for vaccine development and may lead to new therapeutic approaches. We have previously demonstrated the importance of Toll-like receptors (TLRs) in defense against *B*. *pseudomallei* infection [4–6]. Toll-like receptor 5 (TLR5) is a surface receptor of innate immune cells that recognizes flagellin from different bacterial species to initiate host inflammatory responses. In a murine model of respiratory melioidosis, TLR5 plays an important role in host survival [7]. In humans, we previously demonstrated that genetic polymorphism of TLR5 is associated with organ failure and death [8, 9].

Flagellin (FliC) is the subunit protein encoded by *fliC*, which polymerizes to form the filaments of bacterial flagellum that facilitate bacterial motility. *B*. *pseudomallei* flagellin is considered a potential vaccine candidate [10–13]. *B*. *pseudomallei* K96243 FliC consists of 388 amino acids and has a mass of 39,256 Da (http://www.uniprot.org/uniprot/H7C7G3). Since *B*. *pseudomallei* FliC is not commercially available, previous studies have used FliC from *S*. Typhimurium to investigate TLR5 signalling in healthy donors and correlated the outcome in melioidosis patients [9, 14]. In our previous studies, we established a protocol for investigating innate immune responses to various ligands of bacteria [9, 14]. Using the *Salmonella* FliC, we found significant variation in cytokine production among healthy individuals. Since *B*. *pseudomallei* FliC protein sequence shares only 37% similarity with that of *S*. Typhimurium, the activation by *B*. *pseudomallei* FliC with host cells needed to be investigated.

Flagellin of Gram-negative bacteria is not only an immunostimulatory molecule for TLR5 but also a dominant target for the humoral immune response [15]. Data on immune responses to *B*. *pseudomallei* FliC are limited. We previously determined the antibody to rFliC in clinical collections in Thailand and reported that plasma IgG anti-rFliC antibody levels were not significantly different between TLR5 1174C>T in melioidosis cases [8]. During this study, however, we did not determine the association of anti-FliC antibody levels and diabetes or clinical conditions of melioidosis.

The aim of this study was to further investigate human innate and antibody responses to *B*. *pseudomallei* rFliC. Here, we prepared a recombinant *B*. *pseudomallei* rFliC and optimized the conditions for stimulation of HEK-Blue^TM^-hTLR5, THP-1Dual^TM^ and whole blood cells. We determined TLR5-dependent NF-κB activation in HEK-Blue^TM^-hTLR5, THP-1Dual^TM^ cells and compared the levels of IL-1β, IL-6, and TNF-α released from whole blood cells from fourteen healthy individuals after stimulation with rFliC. Furthermore, we used an ELISA to quantitate the IgM and IgG antibody responses to rFliC in serum obtained from different groups of melioidosis patients (diabetes versus non-diabetes, bacteremia versus non-bacteremia and survivors versus non-survivors). It is anticipated that the study of immune responses to *B*. *pseudomallei* flagellin may provide valuable insights toward the potential use of rFliC in the development of vaccines.

## Materials and methods

### Human samples

Fourteen healthy Thai subjects donating blood at the department of Microbiology and Immunology, Faculty of Tropical Medicine, Mahidol University were recruited for participation in the study of cellular response to rFliC. Subjects were between the ages of 25 and 35 and did not report any history of immunodeficiency or inflammatory conditions, chronic diseases, pregnancy in the past six months, anti-inflammatory medication use in the past week, antibiotic use in the past month, vaccination in the past six months, heavy exercise or alcohol consumption in the past 24 h, or smoking in the past month.

Two sets of serum samples were used in this study. The first set was comprised of 45 serum samples from melioidosis patients and 45 serum samples obtained from healthy donors from the same area in northeast Thailand. This serum set was used to determine the different antibody responses between melioidosis and healthy groups by the ELISA as described previously [16]. The second set included serum samples from 200 patients with melioidosis for immunological studies at Sunpasitthiprasong Hospital, Ubon Ratchathani, Thailand as described previously [17, 18]. This set was used to compared the antibody responses among different groups of patients [diabetes (N = 134) versus non-diabetes (N = 66), bacteremia (N = 105) versus non-bacteremia (N = 95), survivors (N = 134) versus non-survivors (N = 64). Diabetes was defined by abnormal HbA1C level. Both male (N = 133) and female (N = 67) patients, aged 18 years or older with melioidosis were enrolled, at a median of 5 days (interquartile range (IQR) 3–6 days, range 2–13 days) after admission. Melioidosis was defined as isolation of *B*. *pseudomallei* from any clinical sample submitted to the laboratory.

### Ethical approval

The study was approved by Ethics Committee of Faculty of Tropical Medicine, Mahidol University (approval number TMEC 17–037, MUTM 2014–079 and MUTM 2012–018), Sunpasitthiprasong hospital (approval number 018/2555), and the Oxford Tropical Research Ethics Committee (reference 64–11). Written informed consent was obtained from for all participants enrolled in the study.

### Preparation of recombinant *B*. *pseudomallei* flagellin protein

rFliC was purified from *E*. *coli* TOP10 (pBpfliC) as previously described [19]. The *fliC* gene (BPSL3319) was PCR amplified from *B*. *pseudomallei* K96243 genomic DNA, cloned into pBAD/HisA (Invitrogen, USA) and expressed in *E*. *coli* after induction by 0.02% L-arabinose. The protein was extracted from cell pellet using B-PER bacterial protein extraction reagent (Thermo Scientific, USA). The soluble protein fraction was then purified for histidine-tagged protein using a Ni-NTA purification system (Invitrogen, USA). The eluate containing rFliC was dialyzed against phosphate buffered saline using 3500 MWCO Slide-A-Lyzer™ Dialysis Cassettes (Thermo Scientific, USA). The rFliC protein was concentrated by 10K MWCO Vivispin concentrator (Sartorius Stedim Biotech GmbH, Germany). The presence of endotoxin in the rFliC was determined by Limulus amebocyte lysate (LAL) assay (Thermo Scientific, USA). The purity of rFliC was verified as a single band protein at molecular weight of 39 kDa by SDS-PAGE and Coomassie blue staining (S1 Fig).

### Cell stimulation assays

HEK-Blue^TM^-hTLR5 cells (Invivogen, USA) were maintained in Dulbecco's Modified Eagle's medium (DMEM) supplemented with 10% heat-inactivated fetal bovine serum (FBS), 100 μg/ml of Normocin (Invivogen, USA), 30 μg/ml of blasticidin (Invivogen, USA) and 100 μg/ml of Zeocin (Invivogen, USA). The cells were incubated at 37°C in 5% CO_2_ in a humidified incubator. The HEK-Blue^TM^-hTLR5 cells were chosen for this study because these cells express only TLR5 on the cell surface. THP1-Dual^TM^ cells (Invivogen) were used to optimize the cell stimulation condition for cells expressing TLR5 and other innate immune receptors [20, 21]. THP-1 monocytic cell line has been shown to express TLR5 [22]. THP1-Dual^TM^ cells were maintained in Roswell Park Memorial Institute (RPMI) 1640 medium supplemented with 10% of FBS and 1% of L-glutamine (Gibco, Invitrogen) at 37°C in 5% CO_2_ in a humidified incubator. Routinely, the cells were passaged every 3 days by seeding at 7 x 10^5^cells/ml in a 75 cm^2^ culture flask. Every other passage, 100 μg/ml of Zeocin and 10 μg/ml of blasticidin were added to the cell culture for maintaining selection pressure.

For cell stimulation assays, HEK-Blue^TM^-hTLR5 at 1.4 x 10^5^ cells/ml were incubated with either rFliC or purified flagellin from *S*. Typhimurium (FLA-ST, ultrapure, Invivogen) at concentrations of 1 ng/ml, 10 ng/ml, and 100 ng/ml in duplicate. THP1-Dual^TM^ cells at 5.6 x 10^5^ cells/ml were stimulated with rFliC or FLA-ST as a control at final concentrations of 100 and 500 ng/ml. The cells were dispensed into the 96-well plate containing 20 μl of 10x stimuli (1,000 or 5,000 ng/ml), incubated at 37°C, 5% CO_2_ for 24 h and then cell supernatants were harvested. To determine NF-κB activation, 200 μl of pre-warmed QUANTI-Blue^TM^ was dispensed into a 96-well plate with 20 μl of the cell supernatant and the plate was incubated at 37°C in 5% CO_2_. Three independent experiments were performed. After incubation for 1 h, SEAP activity in supernatant was assessed by reading the plate at OD 620 nm using a microplate reader (TECAN sunrise, Grӧdig, Austria).

For whole blood stimulation assays, 180 μl of fresh whole blood in heparin mixed 1:1 with RPMI media was added to pre-prepared plates containing 20 μl of stimuli as previously described [9]. For this study, the stimulant was rFliC at a final concentration of 500 ng/ml. Plates with aluminum plate sealer were incubated at 37°C, 5% CO_2_ for 6 h and 24 h. The plates were centrifuged (500 x *g*) prior to collecting the supernatants and then stored at −80°C. IL-6, TNF-α and IL-1β levels were assayed in duplicate using BD OptEIA^TM^ ELISA kit (BD Bioscience, USA).

### ELISA for IgM and IgG antibodies to rFliC

Prior to determining antibody responses to rFliC in patient serum, the optimal conditions for the ELISA were determined using pooled serum from healthy individuals and melioidosis patients as described in previous studies [8, 16, 18]. The optimal concentration of rFliC for coating was 15 μg/ml and the optimal serum dilution was 1:300. These concentrations were used throughout the study. Plasma levels of IgM and IgG antibodies specific to rFliC were determined by rapid Enzyme-Linked Immunosorbant Assays (ELISA) in duplicate as previously described [8, 16, 18]. The secondary antibodies, HRP conjugated rabbit anti-human IgM or IgG (DAKO, Copenhagen, Denmark), were used at dilutions of 1:50 and 1:2000, respectively. ELISAs were developed using TMB substrate (Invitrogen, Camarillo, CA, USA). Results were determined as absorbance value (OD 450 nm) and the average OD values of duplicate wells were used for analysis. Pooled serum from five melioidosis patients and five healthy controls were used as positive and negative controls, respectively.

### Statistical analysis

Statistical analyses were performed using Prism 6 Statistics (GraphPad Software Inc, La Jolla, CA). The Mann-Whitney test was used to test the difference of median OD values between different serum groups and determine the difference of medians of the blood cytokine concentrations. Student *t*-test was used to test differences in means of rFliC-induced NF-κB activation and FLA-ST-induced NF-κB activation. For the study of anti-FliC antibodies in differentiating between melioidosis patients and healthy subjects, a receiver operator characteristic (ROC) curve was created. Areas under the ROC curves (AUROCC) were compared between the performance of ELISA for IgG and ELISA for IgM using a nonparametric method as previously described by DeLong et al. [23]. Differences were considered statistically significant at a p-value ≤ 0.05.

## Results

### Human cellular responses to rFliC

**Fig 1:**
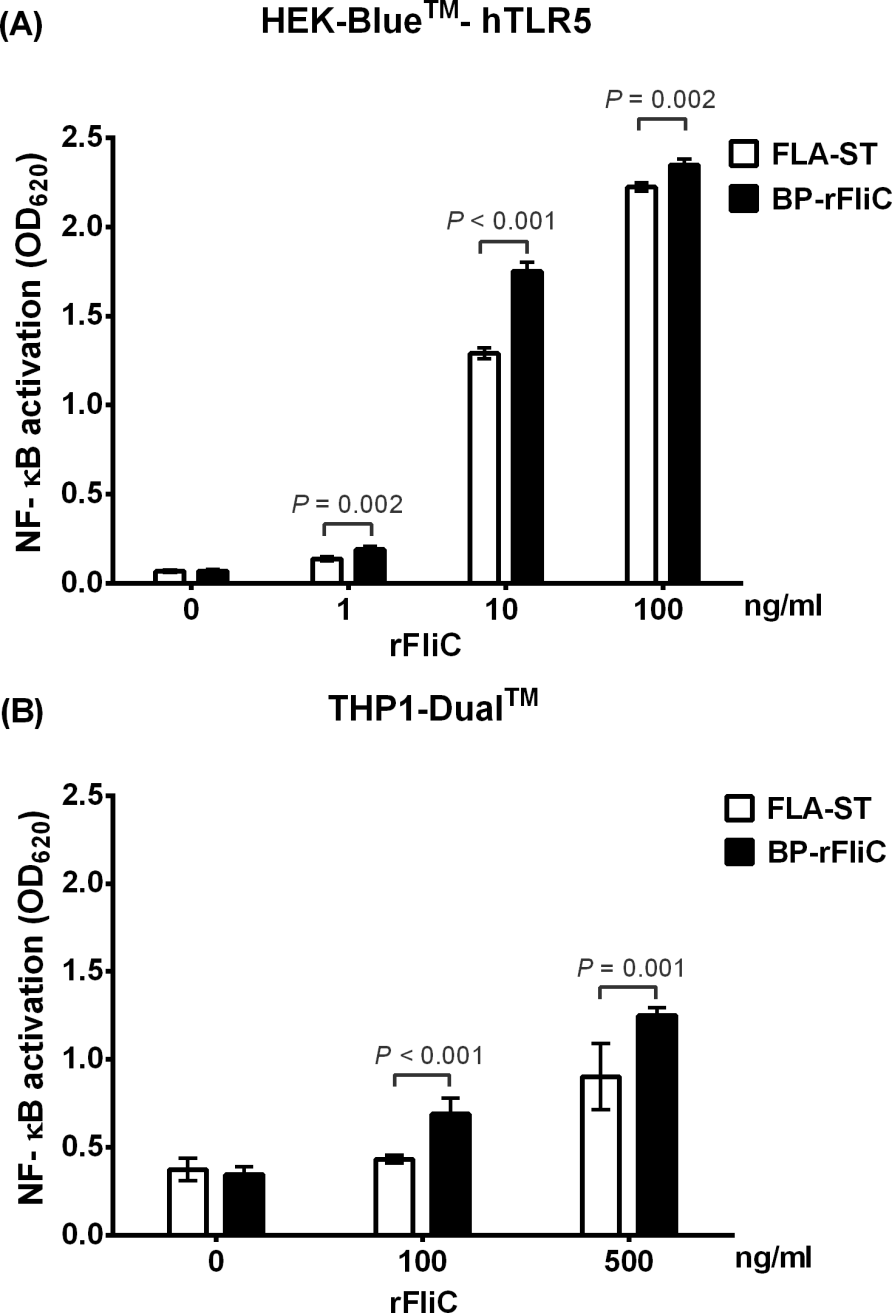
NF-κB activation by rFliC. (A) HEK-Blue^TM^-hTLR5 cells (1.4 x 10^5^cells/ml) in HEK-Blue™ Detection medium were incubated with 1, 10, and 100 ng/ml rFliC in three independent experiments. The cells stimulated with flagellin purified from *S*. Typhimurium (FLA-ST) were used for comparison. After incubation for 24 h, NF-κB activation was determined by monitoring SEAP production. (B) THP1-Dual^TM^ cells (5.6 x10^5^cells/ml) were added into a 96-well plate containing 20 μl of rFliC at indicated concentrations. The supernatant was collected at 24 h after incubation and SEAP activity was measured using the Quanti-Blue assay. The result of THP-1 cell assay was obtained from three independent experiments. Data represent the mean, and error bars represent the standard deviation of the results of three independent experiments conducted in triplicate.

To determine the cellular responses to the purified rFliC, we first stimulated HEK-Blue^TM^-hTLR5 cells with 1, 10 and 100 ng/ml of rFliC for 24 h. The endotoxin concentration of the rFliC was <0.05 EU/mg as determined by LAL assay. The activation of NF-κB was determined by monitoring SEAP levels in cell culture supernatants. Our results demonstrated that rFliC activated NF-κB in a TLR5-dependent manner at concentrations of 10 and 100 ng/ml. We observed a higher NF-κB activation when the HEK-Blue^TM^-hTLR5 cells were stimulated with rFliC compared with those activated with the same concentration of FLA-ST as a control (Fig 1A). These results suggest that *B*. *pseudomallei* rFliC is a potent innate immune stimulator of TLR5.

To further investigate the cellular responses in human monocytes, THP1-Dual^TM^ cells were stimulated with various amounts of rFliC and FLA-ST following which SEAP activity was measured (Fig 1B). In contrast to the results of HEK-Blue^TM^-hTLR5 system, we observed only low levels of NF-κB activation at concentrations of 1 and 10 ng/ml of rFliC in a pilot study. Higher activation levels were detected using concentrations of 100 and 500 ng/ml in dose-dependent manner for both rFliC and FLA-ST. A greater response was observed when cells were stimulated with rFliC compared with those stimulated with FLA-ST at both concentrations. These results suggest that rFliC can activate monocyte inflammatory responses. The HEK-Blue^TM^-hTLR5 cells may express more TLR5 receptors on their surface than the THP1-Dual^TM^ cells.

**Fig 2:**
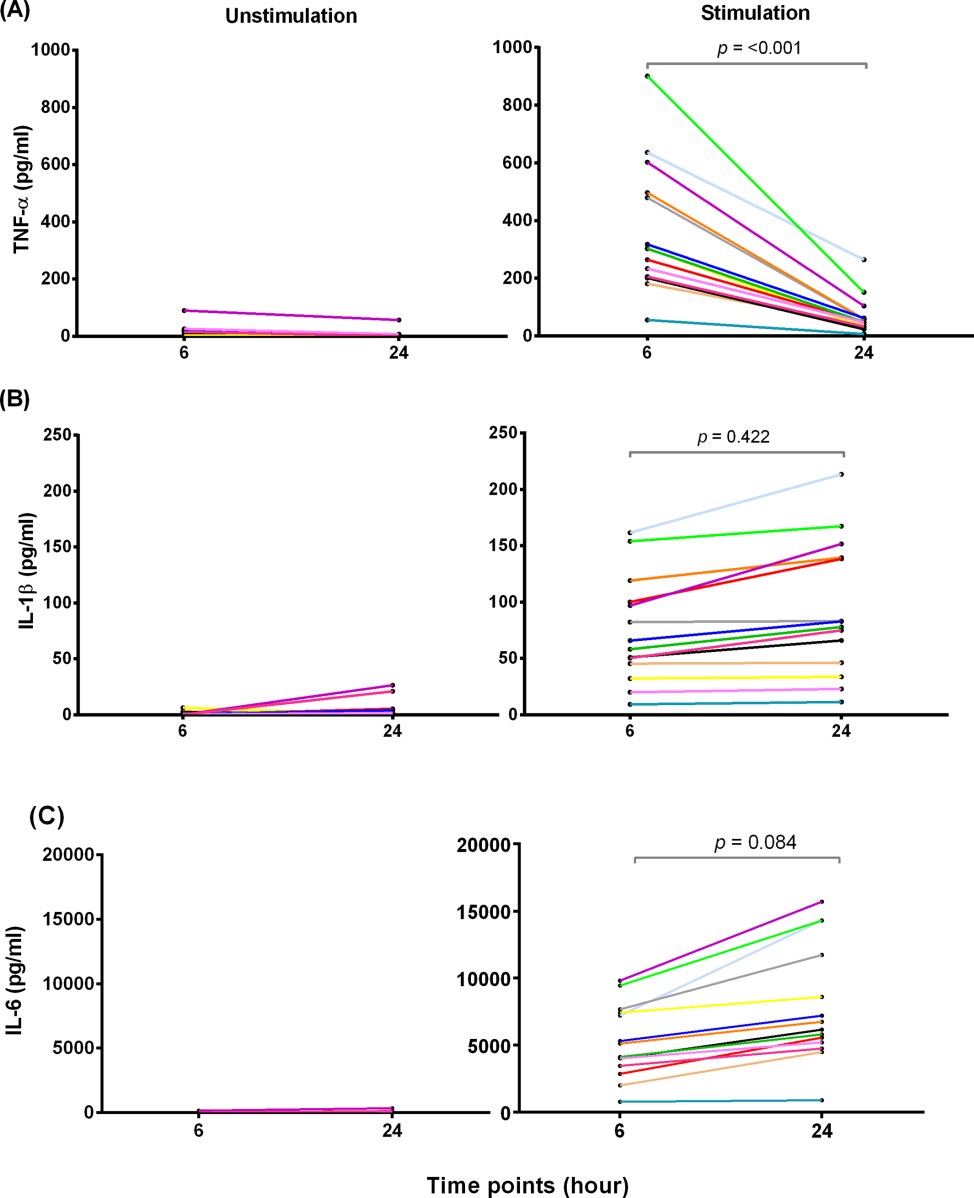
Differential cytokine profiles from individual subjects. Whole blood from healthy subjects (N = 14) was stimulated with rFliC at a final concentration of 500 ng/ml. The supernatants were collected at 6 h and 24 h after incubation and pro-inflammatory cytokine (IL-1β, IL-6 and TNF-α) productions were evaluated by ELISA. Each line represents an individual subject. The differences of medians between 6h and 24h were tested by the Mann-Whitney test.

We examined the possibility of individual differences in responses to rFliC activation by performing whole blood stimulation assays. Blood cells obtained from fourteen healthy donors were stimulated with rFliC at 500 ng/ml and TNF-α, IL-1β and IL-6 releases were measured at 6 h and 24 h (Fig 2A–2C). The TNF-α, IL-1β and IL-6 were produced in response to the rFliC in majority of subject with different kinetics. The results demonstrated different patterns and levels of cytokines among healthy individuals. For all subjects, TNF-α levels were higher at 6 h than at 24 h (median 303.0, IQR 204.0–523.0 pg/ml versus median 48.8, IQR 32.4–72.6 pg/ml, P < 0.001), while the IL-1β levels were similar at 6 h and 24 h (median 62.0, IQR 42.1–105.0 pg/ml versus median 80.4, IQR 43.1–142.0 pg/ml, P = 0.422). In contrast, the level of IL-6 was higher at 24 h compared to 6 h but not statistically significant (median 6445.0, IQR 5090.0–12365.0 versus median 4616.0, IQR 3307.0–7488.0 pg/ml, P = 0.084). We noted that the levels of IL-6 were higher than the other two pro-inflammatory cytokines.

### Human IgM and IgG antibodies to flagellin

**Fig 3:**
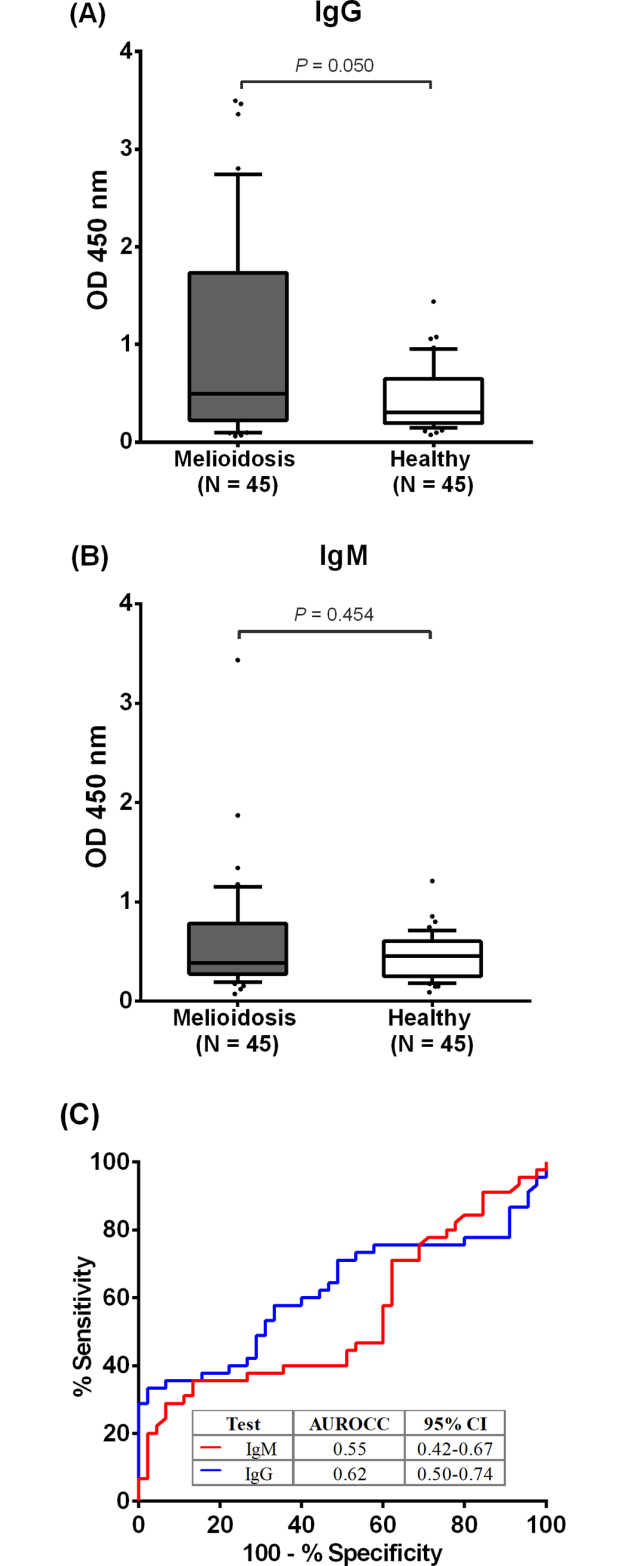
ELISA results of flagellin-specific IgM and IgG antibodies. ELISAs were evaluated for IgM and IgG antibodies using sera from melioidosis patients (N = 45) and healthy donors (N = 45) on the pre-coated plate with 15 μg/ml of rFliC. Box plots show OD at 450 nm of rFliC-specific IgG (A) and IgM (B) in different groups of subjects. All data in box plots are presented as 25^th^ and 75^th^ percentile boundaries in the box with the median line within the box; the whiskers indicate the 10^th^ and 90^th^ percentiles. (C) Receiver Operating Characteristics (ROC) plots.

Our previous studies showed that IgG antibodies that recognize the *B*. *pseudomallei* flagellin protein can be detected in patients with acute melioidosis and plasma anti-FliC antibody levels were not different between survivors and fatal cases [8]. We further compared the IgM and IgG antibody responses in 45 melioidosis patients and 45 healthy donors by ELISA. We observed that the median OD value of ELISA for IgG antibody specific to rFliC was significantly higher in the melioidosis group compared to the healthy group (median OD 0.50, IQR 0.23–1.73 versus median OD 0.30, IQR = 0.19–0.65, P = 0.05) (Fig 3A). However, we did not find a difference for the median OD value of ELISA for IgM (Fig 3B) between these two groups (median OD 0.39, IQR 0.28–0.77 versus median OD 0.45, IQR 0.26–0.60, P = 0.454).

The receiver operating characteristic (ROC) curves of ELISA results from melioidosis patient and healthy donor serum samples were constructed to monitor the shifting on sensitivity and false positive (1-specificity) rates of using antibody to *B*. *pseudomallei* FliC in distinguishing between melioidosis and healthy groups. The area under ROC curve (AUROCCs) for IgM antibody was only 0.55, 95% confidential interval (CI) 0.42–0.67, P = 0.451 and for IgG antibody was 0.62, 95% CI 0.50–0.74, P = 0.050 (Fig 3C). These results suggest that IgG and IgM antibodies against rFliC are not ideal serological markers in differentiating between melioidosis patients and healthy donors.

**Fig 4:**
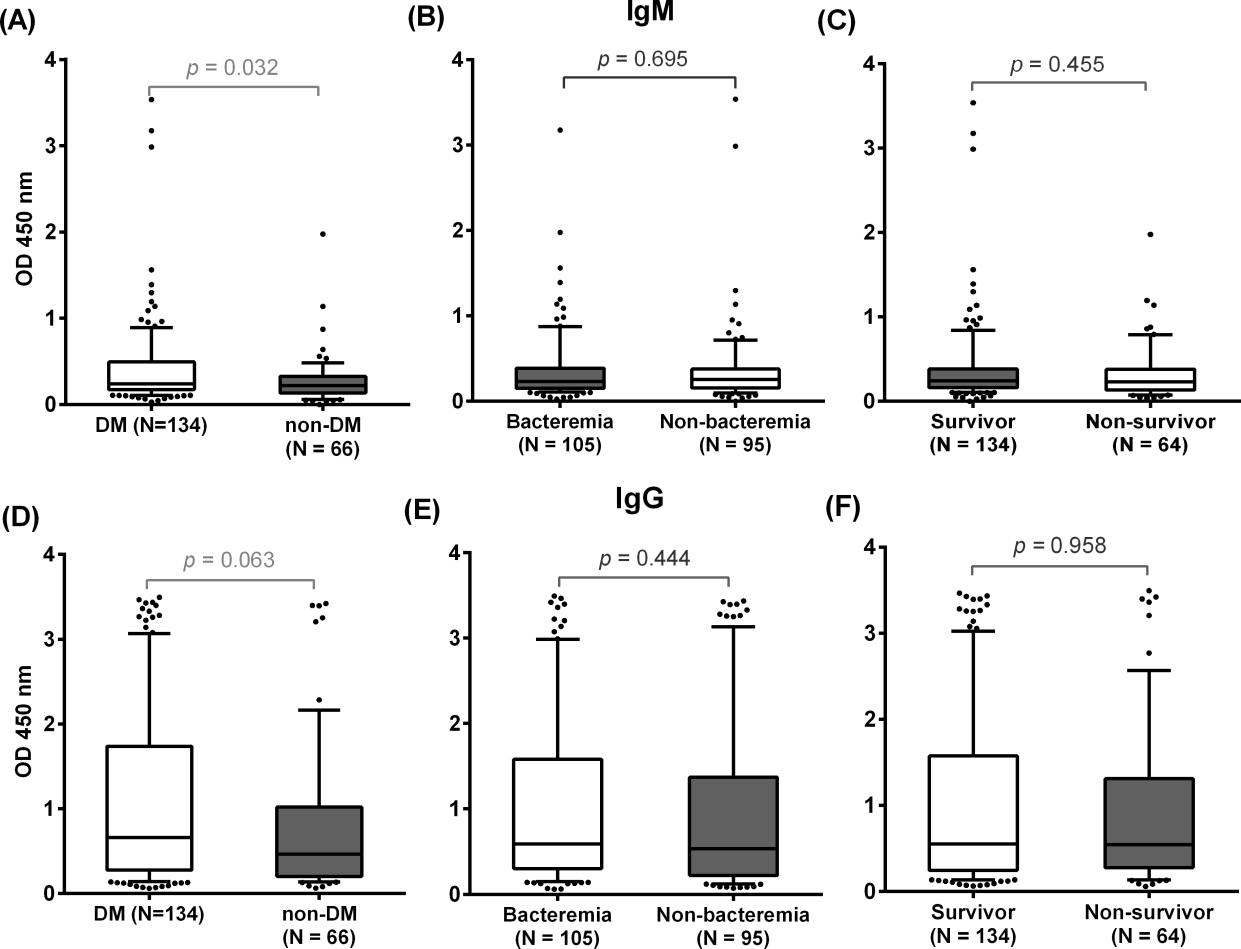
Humoral responses to rFliC. ELISAs using HRP-conjugated rabbit anti-human IgM (A-C) and IgG (D-F) were conducted on prepared plates coated with 15 μg/ml of rFliC. Serum samples from 200 melioidosis patients were used at a dilution of 1:300. Box plots show OD at 450 nm for different groups of patients. All data in box plots are presented as 25^th^ and 75^th^ percentile boundaries in the box with the median line within the box; the whiskers indicate the 10^th^ and 90^th^ percentiles. Mann–Whitney test was used to assess for statistically significant difference of median OD values between different serum groups.

We next examined the effect of diabetes and clinical conditions of the melioidosis patients on the antibody responses to rFliC. In the prospective cohort of 200 melioidosis patients, the median OD values for both IgM and IgG in melioidosis patient serum samples were not different between bacteremia and non-bacteremia groups (for IgM: median OD 0.23, IQR 0.15–0.39 versus median OD 0.26, IQR 0.16–0.38, P = 0.695 and for IgG: median OD 0.59, IQR 0.30–1.58 versus median OD 0.54, IQR 0.22–1.37, P = 0.444) or between survivors and non-survivors (for IgM: median OD 0.24, IQR 0.16–0.38 versus median OD 0.23, IQR 0.13–0.38, P = 0.455 and for IgG: median OD 0.55, IQR 0.24–1.58 versus median OD 0.55, IQR 0.28–1.31, P = 0.958) (Fig 4B, 4C, 4E and 4F). In contrast, the diabetes group had a significant higher IgM antibody (Fig 4A) compared to the non-diabetes group (median OD 0.24, IQR 0.18–0.48 versus median OD 0.22, IQR 0.13–0.33, P = 0.032) while there was no difference of IgG antibody levels between the diabetes and non-diabetes group of melioidosis patients (median OD 0.66, IQR 0.28–1.74 versus median OD 0.46, IQR 0.20–1.02, P = 0.063) (Fig 4D). These data suggest that the patient condition or immune status may affect host immune responses to *B*. *pseudomallei*.

## Discussion

Previous studies have suggested *B*. *pseudomallei* flagellin is a potential vaccine candidate [10–13, 24]. The present study confirmed the previous studies that flagellin is a component of *B*. *pseudomallei* that activates human innate and adaptive immune responses [8, 25–27]. Our observation that rFliC activated HEK-Blue^TM^-hTLR5 cells confirmed that *B*. *pseudomallei* flagellin has TLR5-dependent immunostimulatory function, which is similar to FLA-ST although some differences between the immunoreactive epitopes between rFliC and FLA-ST has been reported. *S*. Typhimurium flagellin (FliC) contains dominant epitopes at residues 339–350 (FliC339-350) and 427–441(FliC427-441) within C-terminal region [28, 29] while dominant immunoreactive epitopes of *B*. *pseudomallei* flagellin are two peptides at positions F51-69 and F270-288 [27].

Our recent study demonstrated that the human body temperature at 37°C facilitates a high growth rate, flagellin gene (*fliC*) expression and maximum motility functions in *B*. *pseudomallei* [30]. These mechanisms and the abundant flagella production may be a prerequisite for acute infection and bacterial dissemination. Flagellin-TLR5 interactions initiate the activation of transcription factor NF-κB and result in secretion of pro-inflammatory cytokines [9, 31–33]. The present study using human whole blood demonstrated that the recombinant *B*. *pseudomallei* flagellin induced pro-inflammatory cytokine (TNF-α, IL-1β, and IL-6) productions. The whole blood stimulations in this study have been observed using a high concentration of rFliC which may not exactly be at physiologically relevant concentrations. However, peripheral leukocytes of patients with acute melioidosis can up-regulate toll-like receptors including TLR5 expression which may enhance the cell signaling [34]. All together, these data suggest that flagellin-TLR5 interactions may have an important role in inflammatory response in melioidosis. The whole blood cytokine responses to flagellin showed different patterns and levels among donors. This may be explained by inter-individual variation effects [14, 35] and may be related to the differences in many host factors, for examples: genetic variation of innate immune receptors such as surface TLR5 and NLRC4 [7], number of cytokine producing cells or other factors. TLR5 and NLRC4 have each been shown to contribute to survival in murine model of respiratory melioidosis [7, 36] and *B*. *thailandensis* infection [37]. It is possible that the different recognition pathways of hosts may be used for flagellin. For example, a previous study used structure-guided mutagenesis to determine the recognition pathways of human and mouse TLR5 and demonstrated species-specific differences in flagellin recognition [38]. These results suggest the importance of different responses to rFliC which may affect host defenses and outcomes from melioidosis. Studies are ongoing to address the influence of TLR5 genetic variation and innate immune responses to *B*. *pseudomallei* rFliC.

Our previous study did not find a relationship between TLR5 genotype and anti-FliC IgG or indirect hemagglutination (IHA) titer during acute melioidosis [8]. This study reports a trend of IgG antibody to rFliC was higher in the melioidosis patient group than the healthy donor group but that IgM responses to flagellin are not significantly different between melioidosis patient and healthy donor groups. Our AUROCC analyses suggest that serum antibodies to rFliC had a low potential value in differentiating melioidosis group from healthy group. IgG and IgM against rFliC were also detected in some healthy controls. Whether this is due to cross-reactivity issues or indicative of previous exposure to *B*. *pseudomallei* remains to be determined. We did not find evidence that antibody responses to rFliC were different between survivors and non-survivors or bacteremia and non-bacteremia patients. These results suggest that natural antibodies against FliC are not correlated with survival and bacteremia in human melioidosis. The activation of TLR5 by flagellin might play a critical role in inflammatory cytokine responses against *B*. *pseudomallei* infection in the initial phase of infection.

Our findings of different flagellin-specific antibody responses between diabetic and non-diabetic patients are of interest. This is concordant with our previous published observations that IgG antibodies to O-polysaccharide (OPS) and hemolysin-coregulated protein 1 (Hcp1) are higher in diabetic than non-diabetic patients [18]. Diabetes is a major risk factor for melioidosis [1–3]. It is possible that diabetic patients may use a different adaptive immune pathway in response to *B*. *pseudomallei* infection than non-diabetes. Another possibility is that diabetic patients may already have raised IgG antibodies due to latent infection. The time of serum sample collection to determine IgG levels may be another factor in the different IgG levels in melioidosis patients enrolled in this study. Our study has performed on the first two weeks of admission but some patients may have clinical symptoms for a long period before visiting the hospital. It is unclear whether this phenomenon only occurs in melioidosis. Further studies are required to address these issues as well as investigate the use of rFliC as a vaccine adjuvant and/or protective antigen.

Roux and colleagues revealed that some *B*. *pseudomallei* strains have a homolog *of lafA* gene encoding a lateral flagellin which is distinct from the polar flagella encoded by *fliC* [39]. The polar and lateral flagellar proteins are different and may have different stimulation effects on host immune cells. It has been shown in *Burkholderia dolosa* that there might be an interaction between the genetic loci encoding polar and lateral flagellin genes as *B*. *dolosa lafA* deletion mutant showed a greater swimming motility than the wild-type due to an increase in polar flagella expression. In addition, the *B*. *dolosa lafA* mutant induced less inflammatory cytokine production by human peripheral blood mononuclear cells and this finding suggests LafA has a role in host immune recognition [39]. However, the role of lateral flagella on host immune response in *B*. *pseudomallei* remains to be investigated.

In summary, our data confirm that rFliC can activate NF-κB activation through the TLR5 pathway. Our results also suggest that the immune responses to *B*. *pseudomallei* flagellin differ among individuals and are influenced by predisposing conditions (e.g. diabetes). IgG antibody responses to *B*. *pseudomallei* flagellin are elevated during acute melioidosis compared to healthy controls but these are not associated with the outcome of infection.

## Supporting information

S1 Fig Purification profile of rFliC of *B*. *pseudomallei* expressed by *E*. *coli* TOP10. The collected fractions along the expression and purification processes were performed SDS-PAGE on 4–12% Bis-tris Bolt gel (10μl/well). The protein fractions were visualized by staining with Coomassie brilliant blue. Lane 1, Protein marker; Lane 2, Non-induced *E*. *coli*; Lane 3, Arabinose-induced *E*. *coli;* Lane 4, Insoluble fraction after treatment with solubilization buffer; Lane 5, Soluble protein after lysis with buffer A; Lane 6, Soluble protein after lysis with buffer B. Lane 7, Soluble protein after treatment with solubilization buffer; Lane 8, Flow-through fraction after applying the soluble protein from treatment with solubilization buffer to the Ni-NTA column; Lane 9, Flow-through fraction after washing; Lane 10, Elution fraction.
(TIF)
