# Supplementary figures and images for: Immune response to recombinant Burkholderia pseudomallei FliC
Source: PLoS One. 2018 Jun 14;13(6):e0198906. doi: 10.1371/journal.pone.0198906 (PMC6002054; doi:10.1371/journal.pone.0198906)

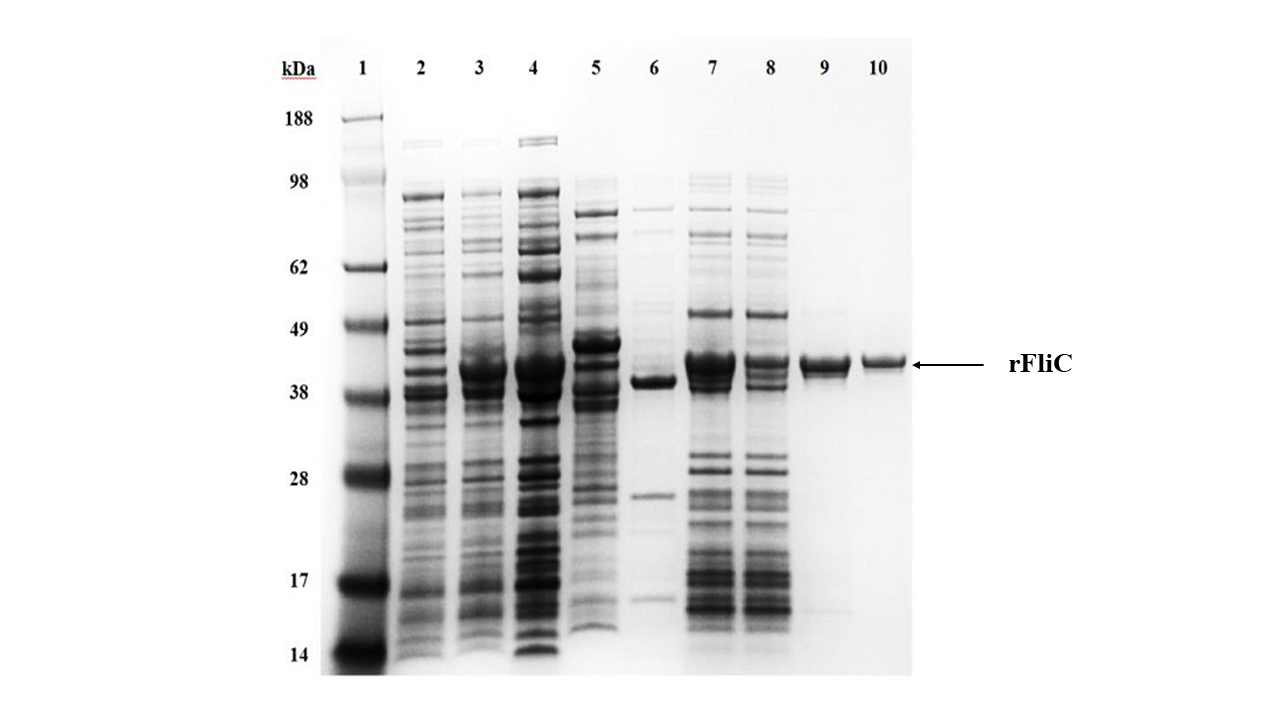

Supplement: S1 Fig — The collected fractions along the expression and purification processes were performed SDS-PAGE on 4–12% Bis-tris Bolt gel (10μl/well). The protein fractions were visualized by staining with Coomassie brilliant blue. Lane 1, Protein marker; Lane 2, Non-induced E. coli; Lane 3, Arabinose-induced E. coli; Lane 4, Insoluble fraction after treatment with solubilization buffer; Lane 5, Soluble protein after lysis with buffer A; Lane 6, Soluble protein after lysis with buffer B. Lane 7, Soluble protein after treatment with solubilization buffer; Lane 8, Flow-through fraction after applying the soluble protein from treatment with solubilization buffer to the Ni-NTA column; Lane 9, Flow-through fraction after washing; Lane 10, Elution fraction. (TIF) [file pone.0198906.s001.tif]
